# Supplementary material for: Beyond native sequence recovery: Improved modeling of the sequence-energy landscape of protein structures
Source: bioRxiv. 2026 Jan 20:2026.01.14.699067. Preprint. [Version 2] doi: 10.64898/2026.01.14.699067 (PMC12871252; doi:10.64898/2026.01.14.699067)
Supplement: Supplement 1 [file NIHPP2026.01.14.699067v2-supplement-1.pdf]

bioRxiv preprint doi: https://doi.org/10.64898/2026.01.14.699067; this version posted January 20, 2026. The copyright holder for this preprint (which was not certified by peer review) is the author/funder, who has granted bioRxiv a license to display the preprint in perpetuity. It is made available under aCC-BY 4.0 International license.

Supplementary Information for
Beyond native sequence recovery: Improved modeling of the sequence-energy landscape of protein structures

Foster Birnbaum, Amy E. Keating

January 15, 2026

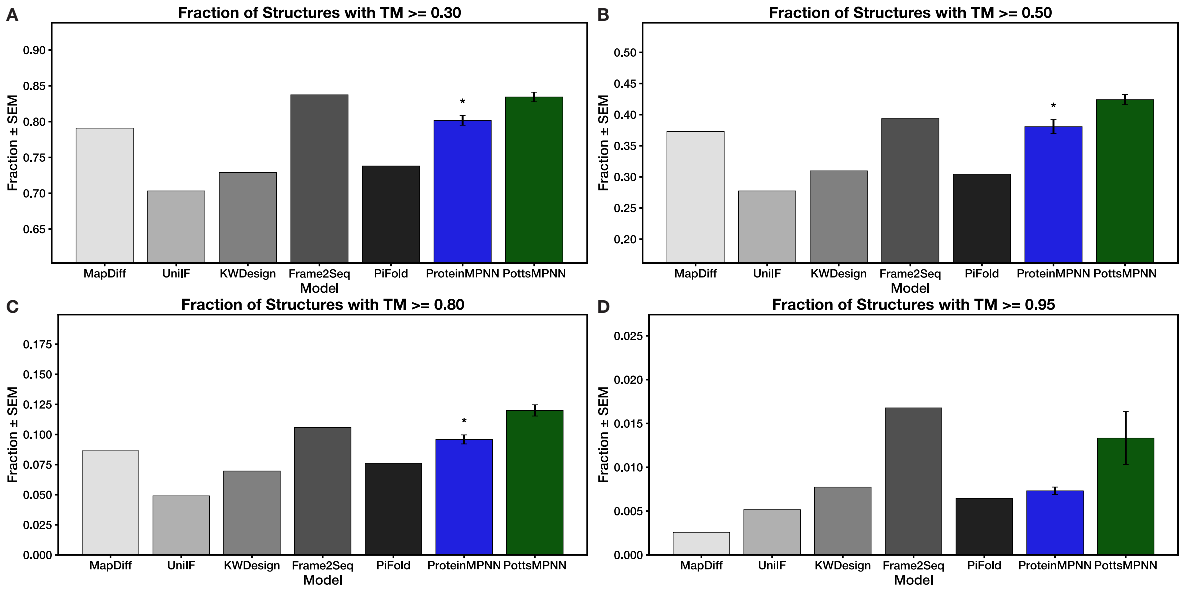

Fig. S1. Expanded TM-score results showing the fraction of generated sequences from the CATH 4.2 test set with AlphaFold2 predicted structures that have TM-scores to the native structures above 0.3 (A), 0.5 (B), 0.8 (C), and 0.95 (D). Error bars show SEM results over three retrained model replicates (where available). Stars indicate statistical significance relative to PottsMPNN, assessed using a two-tailed unpaired t-test over average fractions (* $p < 0.05$; no star indicates non-significance).

bioRxiv preprint doi: https://doi.org/10.64898/2026.01.14.699067; this version posted January 20, 2026. The copyright holder for this preprint (which was not certified by peer review) is the author/funder, who has granted bioRxiv a license to display the preprint in perpetuity. It is made available under aCC-BY 4.0 International license.

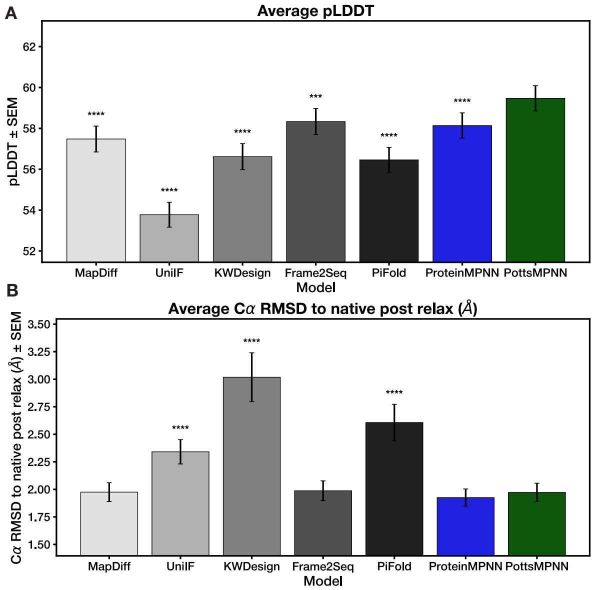

Fig. S2. Structure self-consistency confidence results for benchmark models trained and tested on the CATH 4.2 dataset. (A) AlphaFold2 pLDDT scores for structure predictions of sequences generated using each model. (B) Cα RMSD between the native structures and Rosetta relaxed structures after threading generated sequences onto the native backbone. Error bars show SEM over the proteins in the test set after averaging results over three retrained model replicates. Stars indicate statistical significance relative to PottsMPNN, assessed using a two-tailed paired t-test over per-protein values (* $p < 0.05$, ** $p < 0.01$, *** $p < 0.001$, **** $p < 0.0001$; no star indicates non-significance).

bioRxiv preprint doi: https://doi.org/10.64898/2026.01.14.699067; this version posted January 20, 2026. The copyright holder for this preprint (which was not certified by peer review) is the author/funder, who has granted bioRxiv a license to display the preprint in perpetuity. It is made available under aCC-BY 4.0 International license.

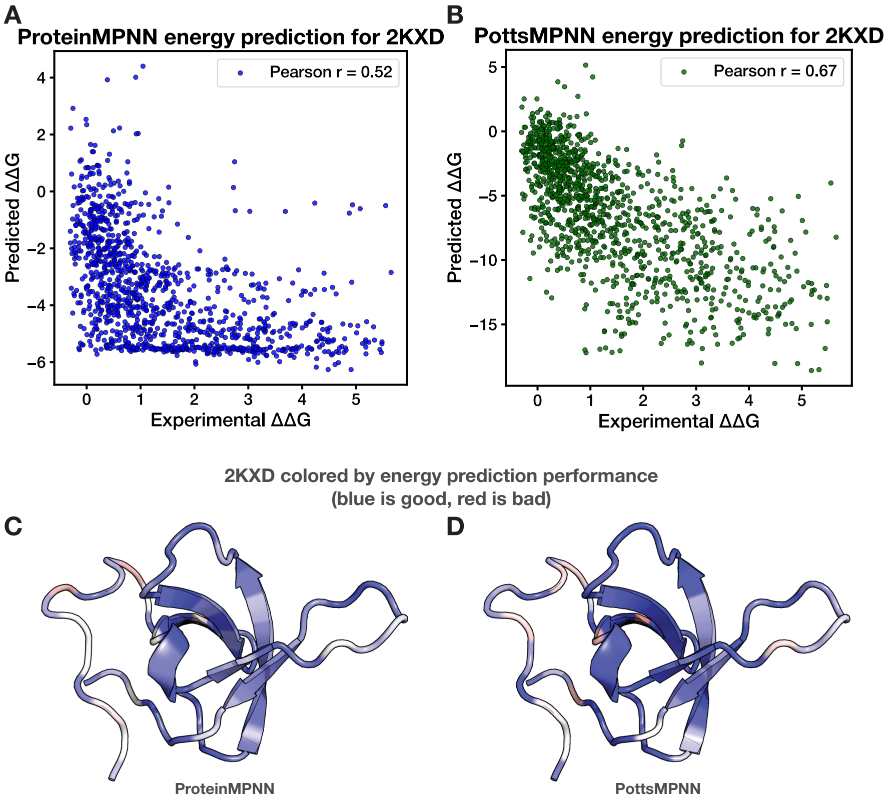

Fig. S3. Case study of using ProteinMPNN and PottsMPNN to predict the effect of single-site mutations on protein stability for 2KXD (1, 2), a protein in the Megascale dataset. (A) ProteinMPNN predictions versus experimental $\Delta\Delta G$ values. (B) PottsMPNN predictions versus experimental $\Delta\Delta G$ values. (C) – (D) Heatmaps showing Pearson r coefficients for the correlation between ProteinMPNN predictions (C) and PottsMPNN predictions (D) against experimental $\Delta\Delta G$ values for mutations at individual sites. Blue indicates a positive correlation, and red indicates a negative correlation.

bioRxiv preprint doi: https://doi.org/10.64898/2026.01.14.699067; this version posted January 20, 2026. The copyright holder for this preprint (which was not certified by peer review) is the author/funder, who has granted bioRxiv a license to display the preprint in perpetuity. It is made available under aCC-BY 4.0 International license.

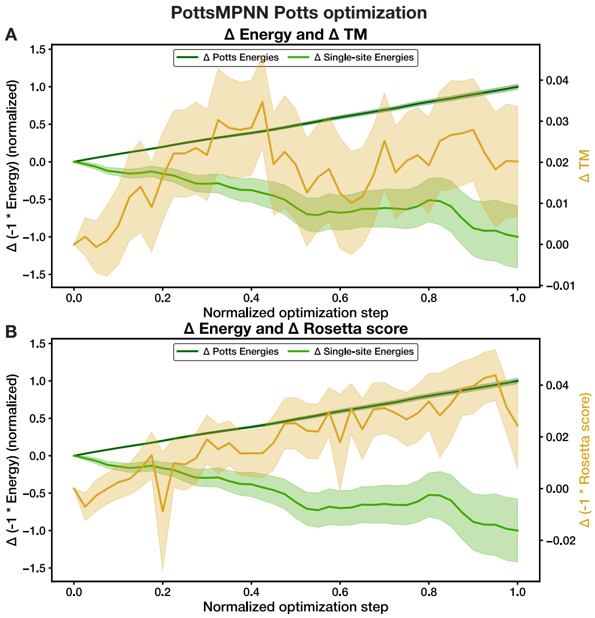

Fig. S4. Tracking sequence-structure self-consistency during optimization of PottsMPNN sequences using the Potts model for 100 random sequences designed for structures from the CATH 4.2 test set. (A) The average normalized change in energy as assessed by the Potts model (dark green) and by the single-site energies (light green) compared to the average change in the TM-score between the native structure and the AlphaFold2 predicted structure (yellow). (B) The average normalized change in energy as assessed by the Potts model (dark green) and by the single-site energies (light green) compared to the average change in the Rosetta score after threading generated sequences onto the native backbone and relaxing (yellow). All scores are plotted such that a higher value is more desirable. Shaded regions indicate the SEM.

bioRxiv preprint doi: https://doi.org/10.64898/2026.01.14.699067; this version posted January 20, 2026. The copyright holder for this preprint (which was not certified by peer review) is the author/funder, who has granted bioRxiv a license to display the preprint in perpetuity. It is made available under aCC-BY 4.0 International license.

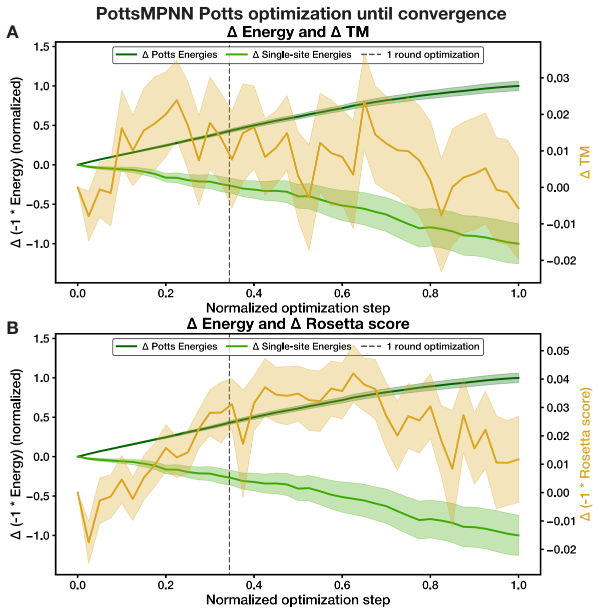

Fig. S5. Tracking sequence-structure self-consistency during optimization of PottsMPNN sequences using the Potts model for 100 random sequences designed for structures from the CATH 4.2 test set until no more favorable mutations are found. (A) The average normalized change in energy as assessed by the Potts model (dark green) and by the single-site energies (light green) compared to the average change in the TM-score between the native structure and the AlphaFold2 predicted structure (yellow). (B) The average normalized change in energy as assessed by the Potts model (dark green) and by the single-site energies (light green) compared to the average change in the Rosetta score after threading generated sequences onto the native backbone and relaxing (yellow). All scores are plotted such that a higher value is more desirable. Shaded regions indicate the SEM. The dotted line indicates the average step at which the standard optimization would have stopped (i.e., after each position in the sequence has been visited once).

bioRxiv preprint doi: https://doi.org/10.64898/2026.01.14.699067; this version posted January 20, 2026. The copyright holder for this preprint (which was not certified by peer review) is the author/funder, who has granted bioRxiv a license to display the preprint in perpetuity. It is made available under aCC-BY 4.0 International license.

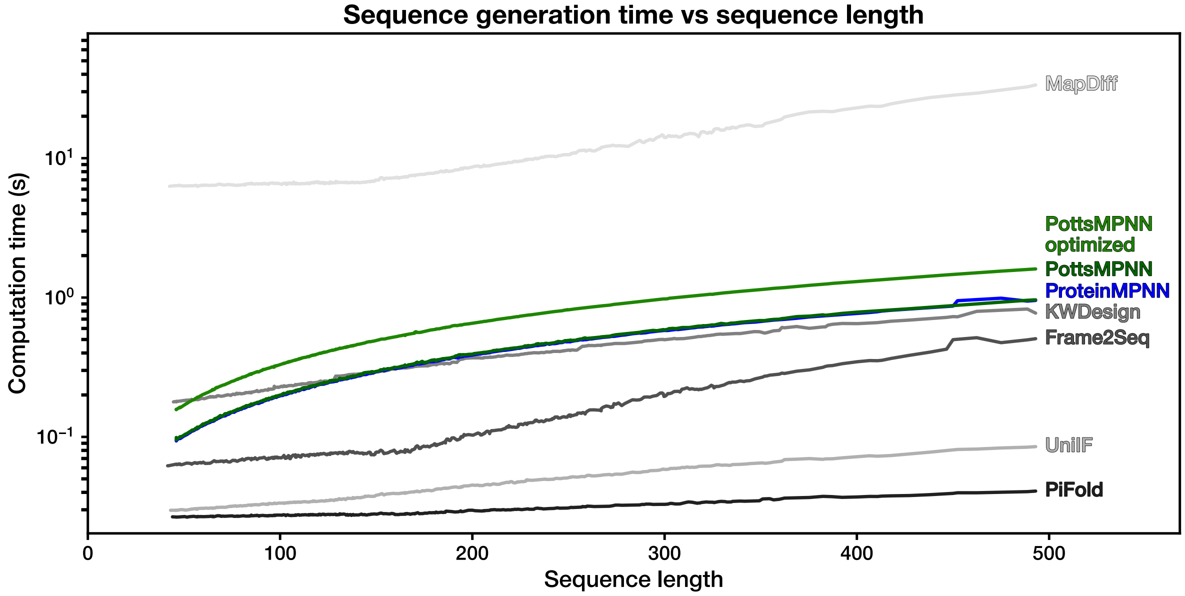

Fig. S6. Time needed to generate 1 sequence for each protein in the CATH 4.2 test set when running the models on an Nvidia A6000 GPU, plotted by sequence length.

bioRxiv preprint doi: https://doi.org/10.64898/2026.01.14.699067; this version posted January 20, 2026. The copyright holder for this preprint (which was not certified by peer review) is the author/funder, who has granted bioRxiv a license to display the preprint in perpetuity. It is made available under aCC-BY 4.0 International license.

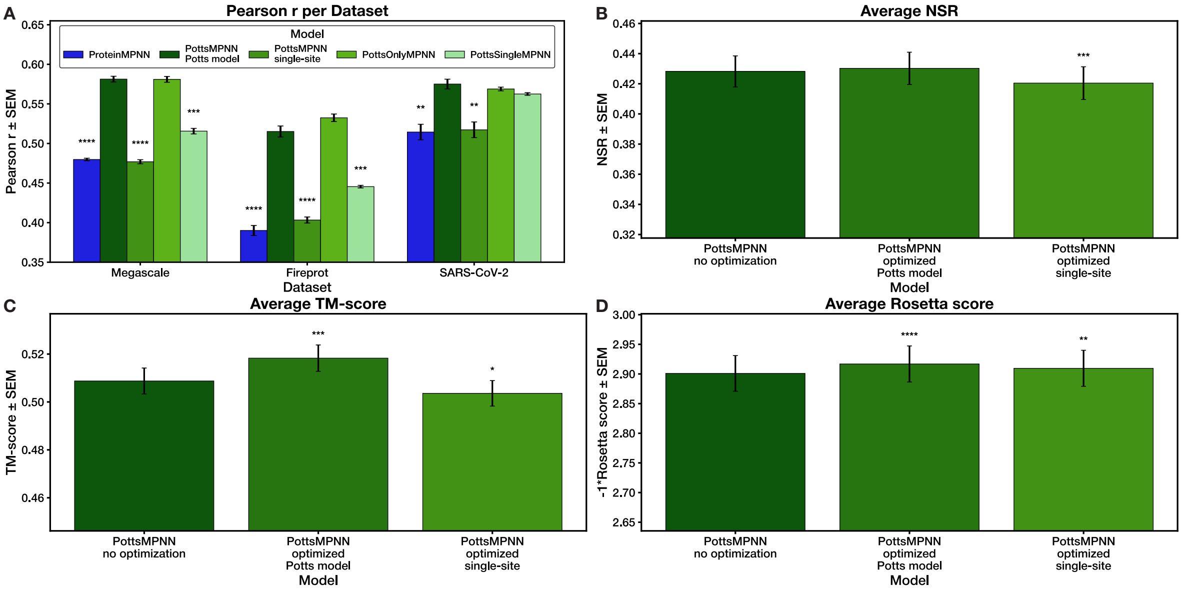

Fig. S7. PottsMPNN Potts model experimentation results for models trained and tested on CATH 4.2. (A) Pearson r for using ProteinMPNN, the Potts model from PottsMPNN, the single-site probabilities from PottsMPNN, PottsOnlyMPNN, and PottsSingleMPNN to predict the effect of single-site mutations on protein stability for three datasets. (B) NSR results for base PottsMPNN (i.e., without optimization), PottsMPNN with one pass of optimization using the Potts model, and PottsMPNN with one pass of optimization using the single-site probabilities. (C) TM-scores between native structures and AlphaFold2 structures predicted for sequences generated using each optimization method. (D) Rosetta scores after threading generated sequences onto the native backbone and relaxing for each optimization method. For (A), error bars show SEM over at least two retrained model replicates. For (B) – (D), error bars show SEM over the proteins in the test set after averaging results over at least two retrained model replicates. Stars indicate statistical significance relative to PottsMPNN Potts model for (A) and to PottsMPNN no optimization for (B) – (D), assessed using a two-tailed unpaired t-test over average Pearson r values for (A) and a two-tailed paired t-test over per-protein values for (B) – (D) and (* $p < 0.05$, ** $p < 0.01$, *** $p < 0.001$, **** $p < 0.0001$; no star indicates non-significance).

bioRxiv preprint doi: https://doi.org/10.64898/2026.01.14.699067; this version posted January 20, 2026. The copyright holder for this preprint (which was not certified by peer review) is the author/funder, who has granted bioRxiv a license to display the preprint in perpetuity. It is made available under aCC-BY 4.0 International license.

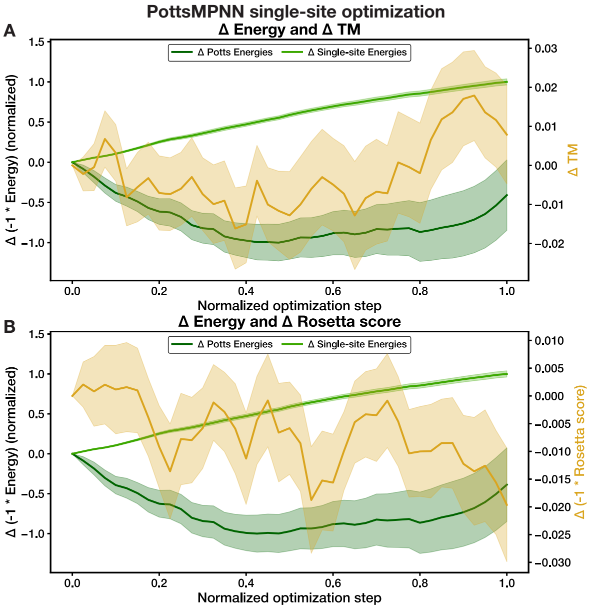

Fig. S8. Tracking sequence-structure self-consistency during optimization of PottsMPNN sequences using the single-site energies for 100 random sequences designed for structures from the CATH 4.2 test. (A) The average normalized change in energy as assessed by the Potts model (dark green) and by the single-site energies (light green) compared to the average change in the TM-score between the native structure and the AlphaFold2 predicted structure (yellow). (B) The average normalized change in energy as assessed by the Potts model (dark green) and by the single-site energies (light green) compared to the average change in the Rosetta score after threading generated sequences onto the native backbone and relaxing (yellow). All scores are plotted such that a higher value is more desirable. Shaded regions indicate the SEM.

bioRxiv preprint doi: https://doi.org/10.64898/2026.01.14.699067; this version posted January 20, 2026. The copyright holder for this preprint (which was not certified by peer review) is the author/funder, who has granted bioRxiv a license to display the preprint in perpetuity. It is made available under aCC-BY 4.0 International license.

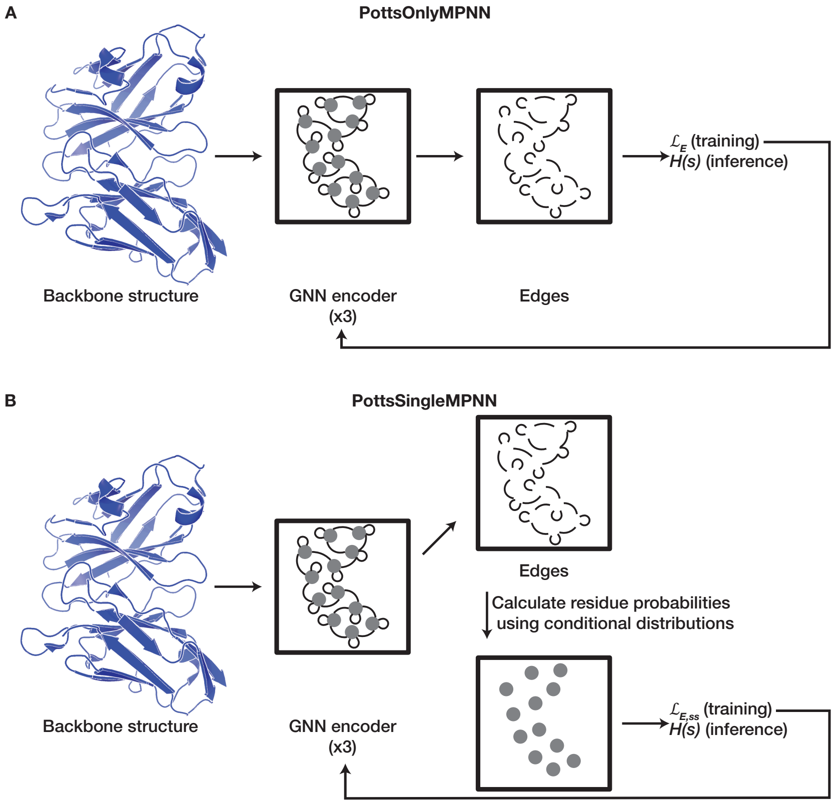

Fig. S9. Overview of the PottsMPNN ablated architectures. (A) Overview of the PottsOnlyMPNN architecture. The graph edges, including self-edges, are supervised to learn single and pairwise residue interaction energies in the form of a Potts model ($H(s)$) that is used to compute structure energies. The Potts model is supervised using a negative log composite pseudo-likelihood loss ($\mathcal{L}_E$) to maximize the probability of native residue pairs. (B) Overview of the PottsSingleMPNN architecture. From the final edge embeddings, conditional probabilities are calculated to generate single-site amino-acid probability distributions, which are supervised by a single-site negative log-likelihood loss ($\mathcal{L}_{E,ss}$); during inference, the Potts model ($H(s)$) is used to compute structure energies.

bioRxiv preprint doi: https://doi.org/10.64898/2026.01.14.699067; this version posted January 20, 2026. The copyright holder for this preprint (which was not certified by peer review) is the author/funder, who has granted bioRxiv a license to display the preprint in perpetuity. It is made available under aCC-BY 4.0 International license.

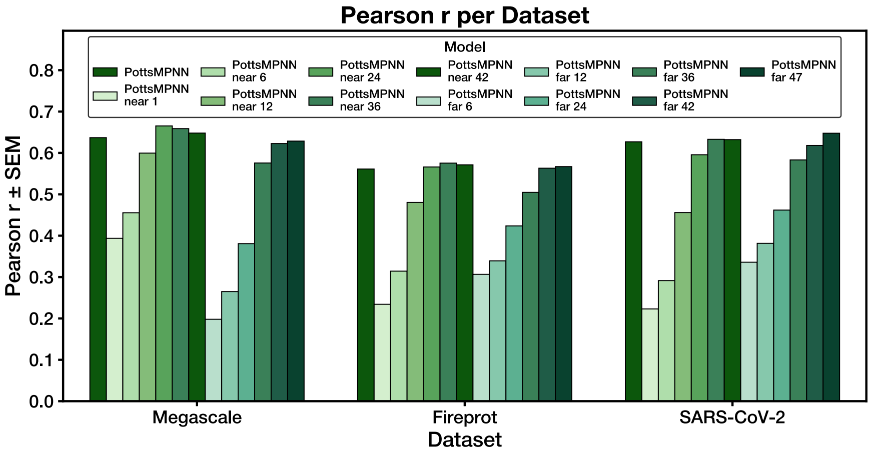

Fig. S10. The impact of including different residue-residue interactions on the Pearson r values for predicting the effect of single-site mutations. Each bar represents the Pearson r resulting from using pair-energies in the Potts model for the indicated neighbor set (e.g., near 6 indicates only the pair-energies with the 6 closest neighbors for each residue were used; far 6 indicates only the pair-energies with the 6 furthest neighbors—out of the 48-nearest neighbors—for each residue were used). Models were trained on PDB-clust.

bioRxiv preprint doi: https://doi.org/10.64898/2026.01.14.699067; this version posted January 20, 2026. The copyright holder for this preprint (which was not certified by peer review) is the author/funder, who has granted bioRxiv a license to display the preprint in perpetuity. It is made available under aCC-BY 4.0 International license.

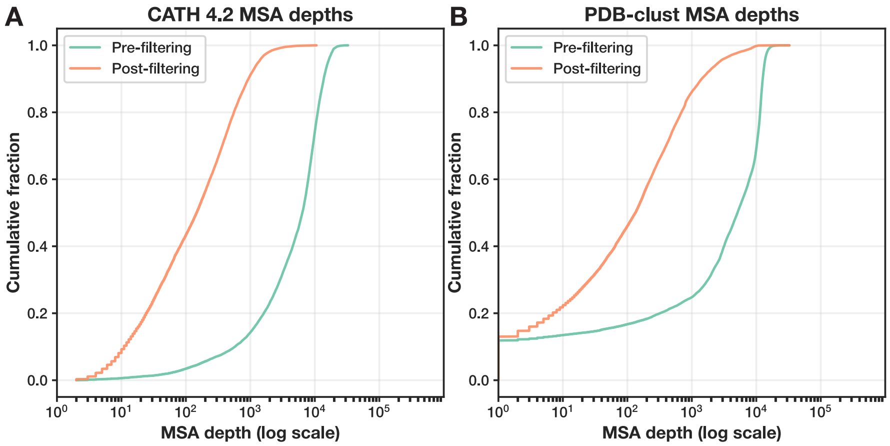

Fig. S11. MSA depths pre- and post-filtering for CATH 4.2 MSAs (A) and PDB-clust MSAs (B). MSAs were filtered using a sequence identity minimum of 50%, a deletion percentage maximum of 20%, and an insertion percentage maximum of 20%. The ∼17% of proteins in the PDB-clust dataset with MSAs of depth 1 correspond to multi-chain proteins for which the paired MSAs are empty.

bioRxiv preprint doi: https://doi.org/10.64898/2026.01.14.699067; this version posted January 20, 2026. The copyright holder for this preprint (which was not certified by peer review) is the author/funder, who has granted bioRxiv a license to display the preprint in perpetuity. It is made available under aCC-BY 4.0 International license.

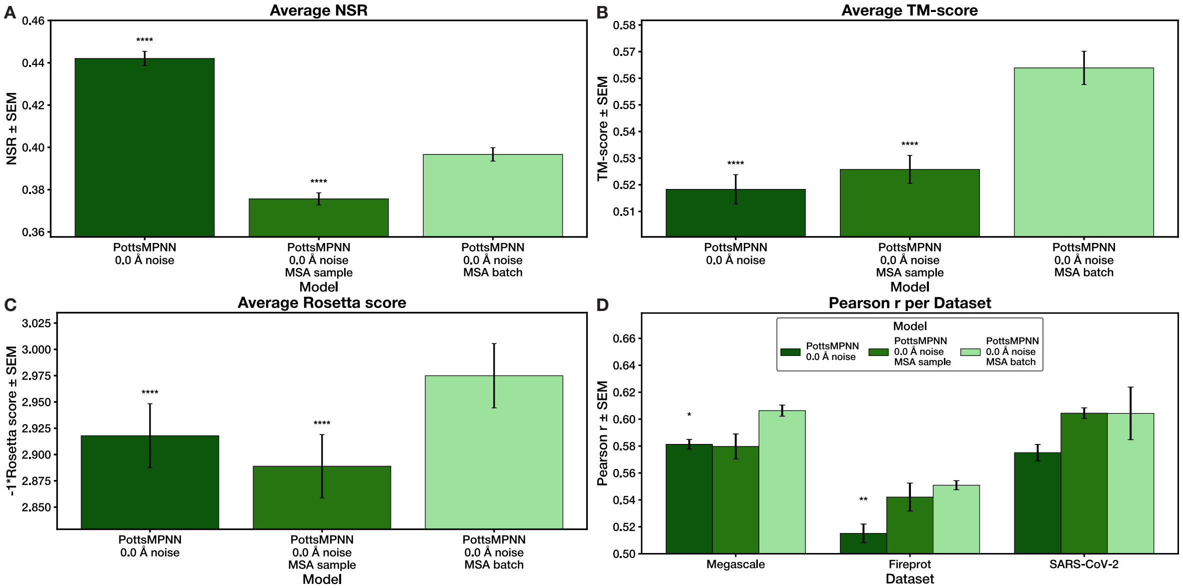

Fig. S12. Using multiple sequences from the MSA during each iteration (MSA batch) versus sampling a single sequence (MSA sample) is key to the improved performance from training with an MSA loss on the CATH 4.2 dataset. (A) NSR results for each model. (B) TM-scores between native structures and AlphaFold2 predicted structures for sequences generated using each model. (C) Rosetta scores after modeling generated sequences on the native backbones for each model. (D) Pearson r for using each model to predict the effect of single-site mutations on protein stability for three datasets. For (A) – (C), error bars show SEM over the proteins in the test set after averaging results over at least two retrained model replicates; for (D), error bars show SEM over at least two retrained model replicates. Stars indicate statistical significance relative to PottsMPNN 0.0 Å noise MSA batch, assessed using a two-tailed paired t-test over per-protein values for (A)–(C) and a two-tailed unpaired t-test over average Pearson r values for (D) (* $p < 0.05$, ** $p < 0.01$, *** $p < 0.001$, **** $p < 0.0001$; no star indicates non-significance).

bioRxiv preprint doi: https://doi.org/10.64898/2026.01.14.699067; this version posted January 20, 2026. The copyright holder for this preprint (which was not certified by peer review) is the author/funder, who has granted bioRxiv a license to display the preprint in perpetuity. It is made available under aCC-BY 4.0 International license.

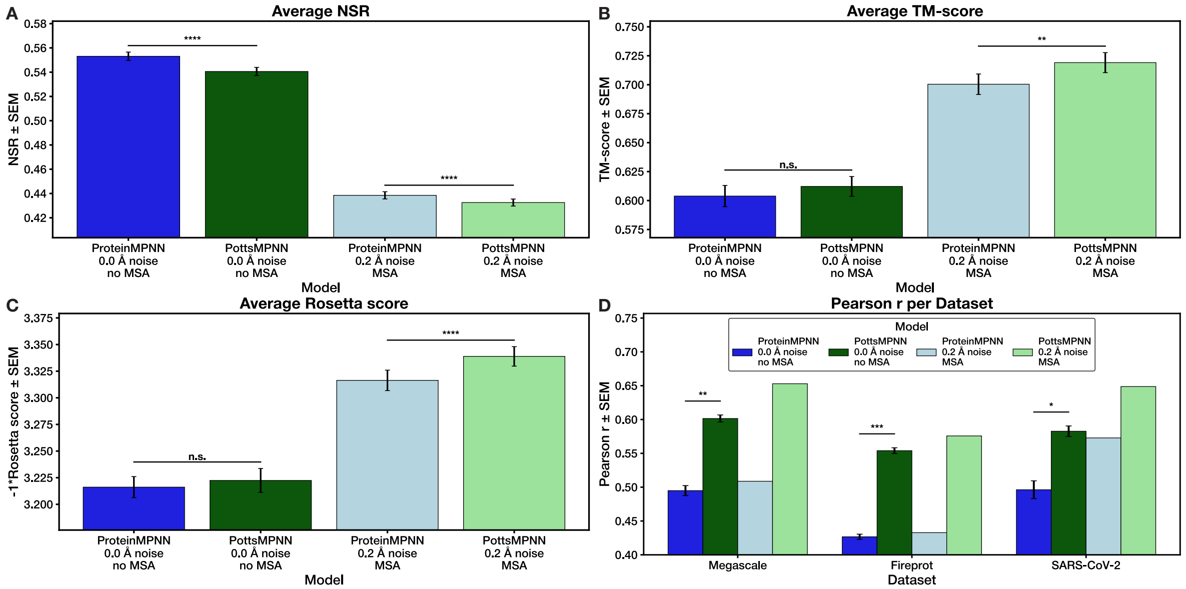

Fig. S13. Training with an MSA loss function on the PDB-clust dataset improves performance. (A) NSR results for each model. (B) TM-scores between native structures and AlphaFold2 predicted structures for sequences generated using each model. (C) Rosetta scores after threading generated sequences onto the native backbone and relaxing for each model. (D) Pearson r for using each model to predict the effect of single-site mutations on protein stability for three datasets. For (A) – (C), error bars show SEM over the proteins in the test set after averaging results over at least two retrained model replicates for the no MSA models; for (D), error bars show SEM over at least two retrained model replicates for the no MSA models. Only one replicate was trained for the MSA models. Stars indicate statistical significance comparing ProteinMPNN to PottsMPNN for each condition, assessed using a two-tailed paired t-test over per-protein values for (A) – (C) and a two-tailed unpaired t-test over average Pearson r values for (D) (* $p < 0.05$, ** $p < 0.01$, *** $p < 0.001$, **** $p < 0.0001$; n.s. indicates non-significance; no bar indicates no statistical test was performed).

bioRxiv preprint doi: https://doi.org/10.64898/2026.01.14.699067; this version posted January 20, 2026. The copyright holder for this preprint (which was not certified by peer review) is the author/funder, who has granted bioRxiv a license to display the preprint in perpetuity. It is made available under aCC-BY 4.0 International license.

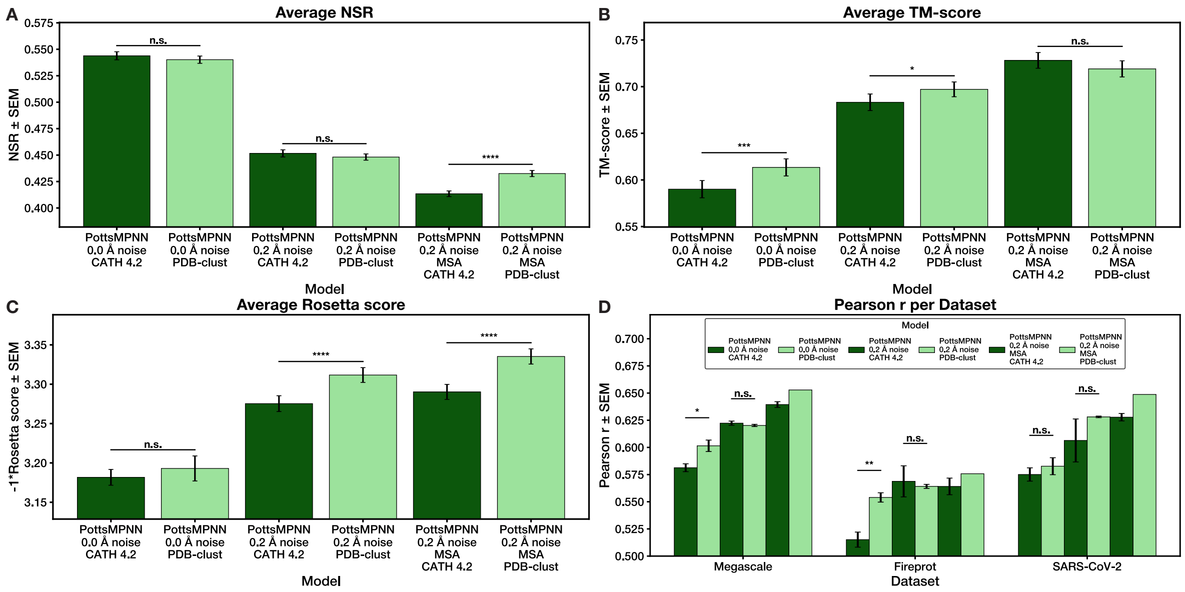

Fig. S14. Training on the PDB-clust dataset is superior to training on the CATH 4.2 dataset when evaluated on a subset of the PDB-clust dataset that is non-overlapping with the CATH 4.2 train set. (A) NSR results for each model. (B) TM-scores between native structures and AlphaFold2 predicted structures for sequences generated using each model. (C) Rosetta scores after modeling generated sequences onto the native backbones. (D) Pearson r for using each model to predict the effect of single-site mutations on protein stability for three datasets. For (A) – (C), error bars show SEM over the proteins in the test set after averaging results over at least two retrained model replicates; for (D), error bars show SEM over at least two retrained model replicates (only 1 replicate of PottsMPNN 0.2 Å noise MSA model was trained on PDB-clust). Stars indicate statistical significance comparing training on CATH 4.2 to training on PDB-clust for each condition, assessed using a two-tailed paired t-test over per-protein values for (A) – (C) and a two-tailed unpaired t-test over average Pearson r values for (D) (* $p < 0.05$, ** $p < 0.01$, *** $p < 0.001$, **** $p < 0.0001$; n.s. indicates non-significance; no bar indicates no statistical test was performed).

bioRxiv preprint doi: https://doi.org/10.64898/2026.01.14.699067; this version posted January 20, 2026. The copyright holder for this preprint (which was not certified by peer review) is the author/funder, who has granted bioRxiv a license to display the preprint in perpetuity. It is made available under aCC-BY 4.0 International license.

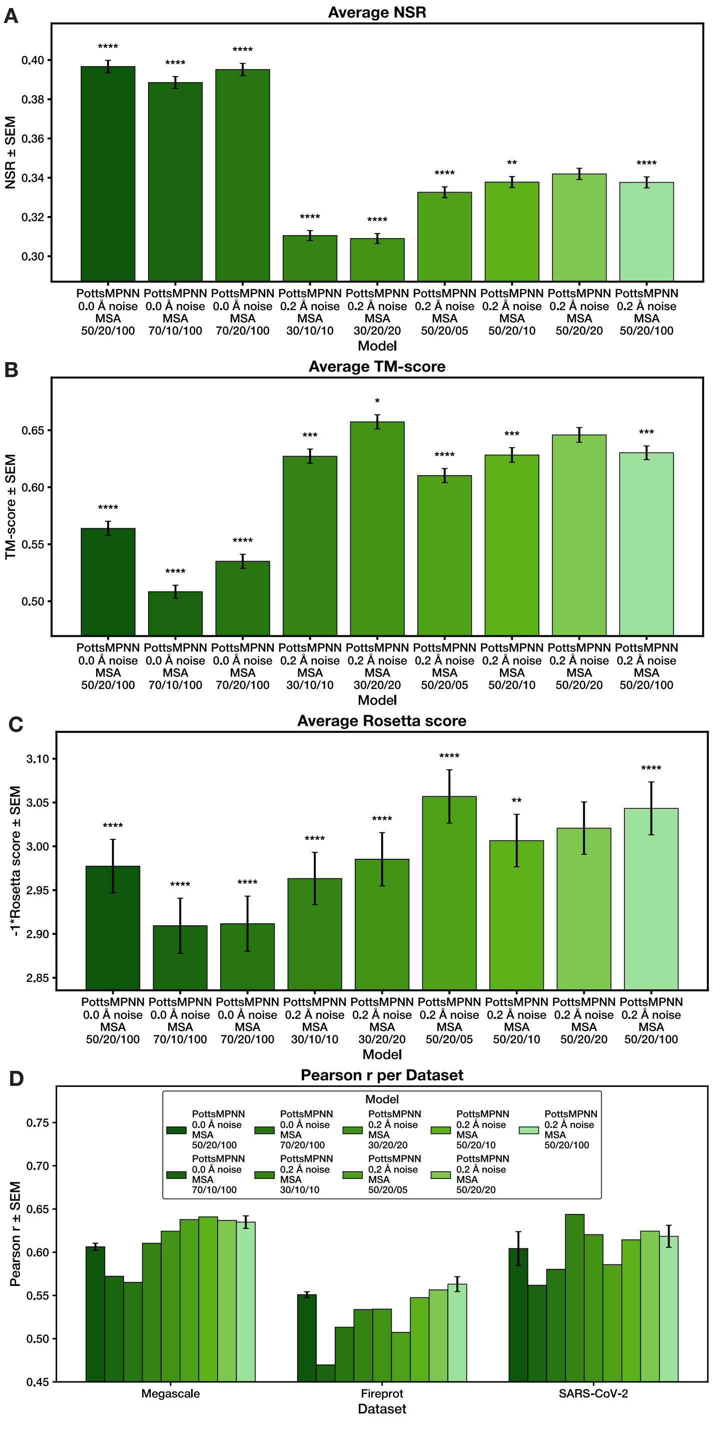

Fig. S15. MSA filtering hyperparameter experiments. (A) NSR results for each model. The hyperparameters are the last line of each label. The first number represents the sequence identity minimum; the second number represents the deletion percentage maximum; and the third number represents the insertion percentage maximum. (B) TM-scores between native structures and AlphaFold2 predicted structures for sequences generated using each model. (C) Rosetta scores after threading generated sequences onto the native backbone and relaxing for each model. (D) Pearson r for using each model to predict the effect of single-site mutations on protein stability for three datasets. For (A) – (C), error bars show SEM over the proteins in the CATH 4.2 test set after averaging over two model replicates; for (D), error bars show SEM over two model replicates. For (D), lack of error bars indicates only one model replicate was trained. Stars indicate statistical significance relative to PottsMPNN 0.2 Å noise MSA 50/20/20, assessed using a two-tailed paired t-test over per-protein values for (A) – (C) and a two-tailed unpaired t-test over average Pearson r values for (D) (* $p < 0.05$, ** $p < 0.01$, *** $p < 0.001$, **** $p < 0.0001$; no star indicates non-significance).

bioRxiv preprint doi: https://doi.org/10.64898/2026.01.14.699067; this version posted January 20, 2026. The copyright holder for this preprint (which was not certified by peer review) is the author/funder, who has granted bioRxiv a license to display the preprint in perpetuity. It is made available under aCC-BY 4.0 International license.

Supplementary References

1. VP Kutyshenko, et al., NMR structure and dynamics of the chimeric protein SH3-F2. *Mol. Biol.* **44**, 948–957 (2010).
2. VP Kutyshenko, et al., The structure of SH3-F2 (2010) https://doi.org/10.2210/pdb2kxd/pdb.
